# Bone Health and Osteoporosis Management in the Elderly: A Review of Guidelines and Orthopedic Implications

**DOI:** 10.7759/cureus.93229

**Published:** 2025-09-25

**Authors:** Samyabrata Das, Vishali Kotwal, Amit Thakur, Loganathan T, Niraj Kumar

**Affiliations:** 1 Department of Orthopaedics, North Bengal Medical College and Hospital, Siliguri, IND; 2 Department of Medicine, Government Medical College, Jammu, IND; 3 Department of Orthopaedics and Traumatology, All India Institute of Medical Sciences, Jammu, Jammu, IND; 4 Department of Footwear Biomechanics Unit, Council of Scientific and Industrial Research (CSIR)Central Leather Research Institute (CLRI), Chennai, IND; 5 Department of Zoology, Laxmi Narain Dubey College, Motihari, Motihari, IND

**Keywords:** aging populations, diagnosis, osteoporosis, rehabilitation, treatment

## Abstract

Osteoporosis, a systemic skeletal disorder characterized by reduced bone mass and structural deterioration, predominantly affects the elderly and leads to increased fracture risk and substantial healthcare burdens. Despite established clinical guidelines, managing osteoporosis in older adults remains challenging due to comorbidities, polypharmacy, and variable guideline adherence. This review aims to critically examine current osteoporosis management strategies, with a focus on guideline recommendations, diagnostic tools, pharmacological and non-pharmacological interventions, and orthopedic implications in the elderly population. A comprehensive literature search of recent high-quality studies and clinical guidelines was conducted, emphasizing evidence-based practices in screening, treatment, and rehabilitation. Key findings reveal that while tools like Fracture Risk Assessment Tool (FRAX) and Dual-energy X-ray Absorptiometry (DXA) remain central to fracture risk assessment, emerging imaging modalities and digital health technologies offer enhanced diagnostic precision. Nutritional supplementation, tailored exercise, and pharmacotherapy, including novel anabolic agents, form the cornerstone of effective fracture prevention. The orthopedic management of fragility fractures requires specialized surgical techniques and multidisciplinary rehabilitation to improve functional outcomes. Challenges include medication adherence, managing comorbidities, and integrating personalized care. The review highlights the need for a holistic, interdisciplinary approach combining medical, surgical, and rehabilitative care, supported by patient education and technological advancements. Optimizing osteoporosis management in the elderly is crucial to reducing fracture incidence, minimizing morbidity, and enhancing quality of life in aging populations.

## Introduction and background

Osteoporosis involves the whole skeleton and is characterized by a loss of bone mass and changes in the bone’s structure, which increases the chance of broken bones [1]. It is most common among the elderly, making it a major public health issue around the world [2]. The loss of bone strength in aging comes from the imbalance between bone breakdown and bone growth [3]. As a result of this decline, along with risks from within and outside the body, the chance of hip, vertebral, and wrist fractures rises [4]. Osteoporosis is much more common in older people, especially in postmenopausal women, although men are also greatly affected [5]. Epidemiology shows that about one-third of women and one-fifth of men over 50 will have an osteoporotic fracture during their lives [6]. As a result, these fractures can lead to long hospital stays, a loss of independence, and a death rate of up to 20% within one year [7]. Because symptoms of vertebral fractures are often mild or vague, they are often not noticed early and can cause ongoing pain, changes in the spine, and breathing problems, significantly reducing a person’s quality of life [8]. Healthcare systems are under great pressure because acute care, rehabilitation, and long-term support are costly [9].

Maintaining bone health in seniors is necessary to prevent fractures and allow them to remain independent. Bone is constantly being remodeled by adding and removing parts, but as we age, resorption increases more than formation, causing bone loss [3,10]. A lack of estrogen in women, not having enough calcium and vitamin D in the diet, reduced exercise, and common health problems can all make this problem worse [11]. Men are more likely than women to be diagnosed with osteoporosis later in life, so doctors should provide different approaches for each group [12]. Apart from fractures, osteoporosis also causes ongoing pain, limits movement, and leads to depression and social isolation, which all reduce quality of life and increase the need for support from caregivers [8,9]. Economically, osteoporosis imposes a growing strain on healthcare resources. Because the elderly population is growing, the costs of treating and caring for fractures are anticipated to increase [2,9]. As a result, we must focus on effective prevention, prompt diagnosis, and complete treatment strategies.

In recent years, numerous clinical guidelines have emerged to standardize the screening, diagnosis, and treatment of osteoporosis. The guidelines mention using tools for assessing risk, various medicines, changes in lifestyle, and ways to prevent falls [11,12]. Even so, it is difficult to apply these guidelines to elderly patients who have many health problems [12]. Because of the quality of the bones in fragility fractures, orthopedic care for these injuries involves unique surgical methods and rehabilitation plans to help patients recover well [4,7]. This review compiles current information on osteoporosis in the elderly, paying special attention to how it can be managed and what orthopedic issues may arise. It closely examines current recommendations, highlights practical difficulties, and emphasizes the need for a combined approach that incorporates both medical and surgical care. The goal of this review is to help clinicians, orthopedic surgeons, and healthcare policy makers find ways to improve bone health and decrease the risk of osteoporotic fractures in older people.

**Figure 1:**
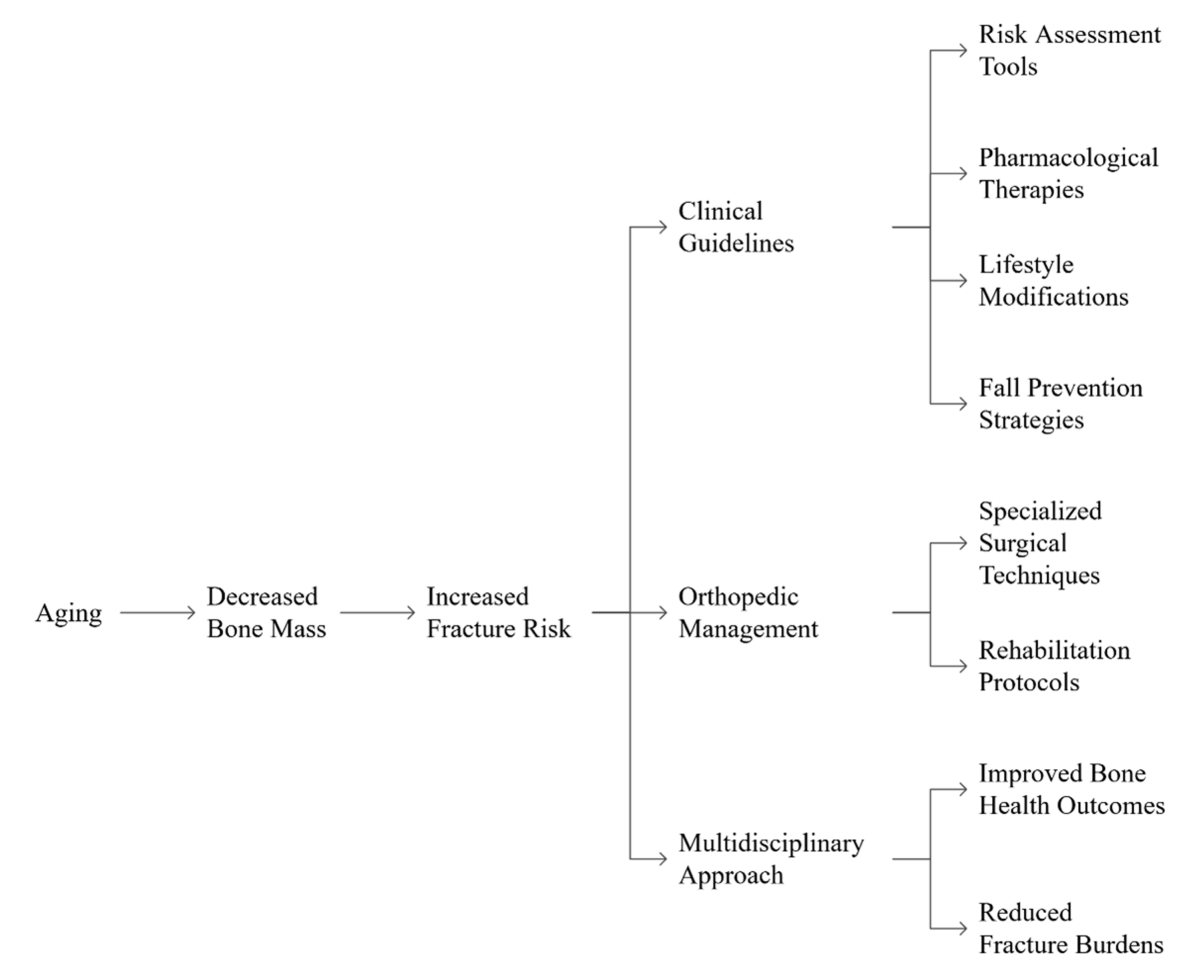
Conceptual framework of osteoporosis management in the elderly Image credit: Loganathan T

Figure 1 shows the progression from bone mass loss due to aging, which increases the risk of fractures, to various interventions like using clinical guidelines, orthopedic care, and a team-based approach. The use of risk assessment tools, various medications, lifestyle changes, surgical techniques, and rehabilitation programs is described to highlight how different approaches can improve bone health and lower the number of fractures in elderly people.

Objectives of the review

This review aims to critically appraise and synthesize contemporary guidelines on screening/diagnosis, pharmacologic management, secondary fracture prevention, and rehabilitation for osteoporosis in adults ≥65 years. We highlight strengths and limitations, assess the evidence underpinning key recommendations, and identify practice gaps and research priorities.

Methodological considerations

Several electronic databases were searched, including PubMed, Scopus, and the Cochrane Library, to find studies, guidelines, and reviews related to osteoporosis and its effects on orthopedics in the elderly. Researchers used both Medical Subject Headings (MeSH) and keywords that matched bone health, osteoporosis, elderly people, fracture care, and clinical guidelines. Only studies published in English over the last 15 years were reviewed, with preference given to those that were high quality, such as randomized controlled trials, systematic reviews, meta-analyses, and clinical practice guidelines. Articles were eligible if they included adults aged ≥60 years and addressed osteoporosis diagnosis, treatment, or management; studies were excluded if they involved children or animals, focused on non-osteoporosis bone disorders, were basic science or in-vitro research, conference abstracts without full text, editorials/opinions, case series with fewer than 10 participants, or did not provide data for adults aged ≥60 years. Key findings, recommendations, and clinical implications were gathered from the data in a standard way and then combined into a narrative to help understand the main ideas. Potential problems with this approach are publication bias, working only with English-language studies, and the fact that not all studies are designed equally, which can make it difficult to generalize. Even with these limitations, the approach was designed to present a reliable and balanced summary of the latest findings on elderly bone health and fracture management.

## Review

Pathophysiology of osteoporosis in the elderly

Bone remodeling unnecessarily occurs, as osteoclasts remove old bone and osteoblasts build new bone. As a result of this cycle, bones stay strong and minerals are balanced [13]. In adults with healthy bones, bone breakdown and growth are closely connected, helping to keep the bones strong [14]. But as we get older, our bones lose this balance, causing bone loss and changes in their structure, which are major features of osteoporosis [15]. With age, changes in the body lead to a weakening of bones. One of the main reasons is fewer active osteoblasts. This reduces the amount of bone that can be formed [16]. At the same time, the activity of osteoclasts usually remains the same or goes up, which causes bone mass to decrease [17]. Moreover, bones become more fragile and less able to effectively handle stress due to changes within the bone matrix through increased collagen cross-linking and micro damage [18]. Hormonal alterations significantly influence this process. A lack of estrogen in postmenopausal women leads to increased bone loss by helping osteoclasts live longer and work harder [19]. Bone loss in aging men is partly due to the slow decrease in the awkward imbalance of estrogen to testosterone [20]. Reduced calcium absorption, inadequate vitamin D, and kidney problems make the bone loss worse for elderly people [21].

In addition, certain health issues that affect older adults make these changes more serious. Chronic inflammation, common in aging, stimulates osteoclastogenesis [21]. The presence of diabetes, rheumatoid arthritis, or long-term use of corticosteroids negatively influences bone remodeling [20]. A lack of exercise and sarcopenia also decrease the pressure on bones, which is necessary to preserve their strength through mechanotransduction [16]. As a result, osteoporosis develops in the elderly due to a variety of factors, including reduced bone growth, imbalance in bone remodeling, chronic diseases, and hormonal issues. To develop effective ways to reduce fracture risk and maintain healthy bones, we must first understand these mechanisms [15]. Table 1 lists the main features, validated groups, and limitations of FRAX, QFracture, and DXA, noting their advantages and disadvantages.

**Table 1:** Comparison of major osteoporosis risk assessment and screening tools

Screening Tool	Clinical Inputs	Validated Populations	Limitations	References
FRAX (Fracture Risk Assessment Tool)	Age, sex, BMI, prior fracture, glucocorticoid use, smoking, alcohol intake, secondary osteoporosis; optional femoral neck BMD	Global (many countries)	Does not include fall history; may underestimate risk in some ethnic groups.	[15]
QFracture	Age, sex, BMI, comorbidities (e.g., rheumatoid arthritis, diabetes), medication use, smoking, alcohol use	UK population	Less validated outside the UK; complexity may limit use	[9]
Dual-energy X-ray Absorptiometry (DXA)	Bone mineral density at the hip and lumbar spine	Global, all adults	Does not assess bone quality; limited accessibility in some regions	[11]
Other Tools (e.g., QUS, OST)	Various risk factors, sometimes without BMD	Limited or regional validation	Less accurate than FRAX or QFracture; not widely recommended	[19]

Risk assessment and screening guidelines

Identifying and managing osteoporosis early is important, and this is best done by effective risk assessment and screening in elderly people, who are more likely to have fractures [22]. Several tools have been proven to work by using clinical factors and sometimes bone mineral density (BMD) to predict a person’s risk of fracture. FRAX is one of the most popular tools used around the world. It estimates the 10-year risk of hip and major osteoporotic fractures using clinical factors such as age, sex, body mass index, history of previous fractures, glucocorticoid use, smoking, alcohol intake, and other causes of osteoporosis [23]. FRAX can be applied either with femoral neck BMD, which increases accuracy by incorporating bone density into fracture risk estimates, or without BMD, relying solely on clinical risk factors when DXA scanning is unavailable or impractical [24]. In the United Kingdom, QFracture is widely used, incorporating comorbidities and medication use into the risk assessment [25].

Most guidelines suggest that screening should be offered to older adults, especially postmenopausal women and men who are 70 years or older [26]. It is often advised to screen people earlier if they have had a fragility fracture, are on corticosteroids for a long time, or have problems with bone metabolism [27]. The guidelines suggest assessing individuals based on sex and ethnicity, since fracture risk varies among different populations [26]. Currently, dual-energy X-ray absorptiometry (DXA) is the main method used to diagnose osteoporosis. Bone density is measured by DXA, mainly at the hip and lumbar spine, and is important for confirming the diagnosis and following treatment progress [24]. Yet, since BMD does not include every risk factor for fractures, using FRAX with BMD improves the decision to start treatment [23].

Nutritional and lifestyle interventions

Making changes to the diet and lifestyle is necessary for keeping your bones healthy and avoiding fractures as you get older [28]. Calcium and vitamin D are key supplements because they are important for bone health and strength [29]. A low calcium intake can lead to faster bone loss [30]. Both the National Osteoporosis Foundation (NOF) and the International Osteoporosis Foundation (IOF) suggest that adults over 50 should aim for 1,000 to 1,200 mg of calcium daily, either through food or supplements [31]. Vitamin D helps the gut absorb calcium and controls how osteoblasts and osteoclasts work to remodel bones [19]. Most experts recommend that elderly individuals take 800 to 1,000 IU of vitamin D daily, especially if they do not get enough sunlight or have a documented deficiency [11]. Good vitamin D levels help muscles work better, which reduces the risk of falls and broken bones [32]. A proper diet for bone health includes foods that are rich in calcium, vitamin D, protein, magnesium, phosphorus, and vitamin K [22]. Dairy, leafy greens, fatty fish, and foods fortified with all sources that help you meet these needs. Adequate protein is essential for both maintaining bone structure and preventing loss of muscle mass, which is important for fighting sarcopenia and strengthening the skeleton [28].

Physical activity, combined with nutritional support, helps build bones and improve muscle and nerve function [33]. Walking, jogging, and resistance training place stress on bones, which is important for their strength [33]. Strength and balance exercises done at least two to three times a week improve your balance and lower the risk of falling [32]. To prevent falls, people should modify their homes, use glasses if needed, have their medications reviewed, and use assistive devices when appropriate [32]. Teaching patients how to move safely and avoid falls adds to the risk reduction process [3]. Although supplementation is well known to help, some recent studies suggest we should not give supplements to everyone, but use an individualized approach [29]. Proper nutrition, suitable exercise, and fall prevention measures support bone health and reduce fractures in seniors [18].

Pharmacological management

Treating osteoporosis with medication is very important for reducing the risk of fractures and improving bone strength in older people at high risk [34]. When deciding on a therapy, doctors must weigh its effectiveness, safety, patient preference, and conditions that might prevent its use [35]. Although bisphosphonates and denosumab both help reduce fractures, they differ in how they are given, the side effects they cause, and who can use them safely [34]. For patients with severe osteoporosis or those who did not respond to antiresorptive medicines, new anabolic therapies are available [6].

A major issue in clinical care is ensuring patients take their osteoporosis medications correctly. Medication adherence is affected by side effects, complicated dosing, and difficulty in understanding why the medication is needed when the patient believes they are asymptomatic [35]. Regular tests of bone density and check-ups by the doctor are necessary to check if the treatment is effective and safe, so that any necessary changes can be made promptly [36]. In certain cases, a temporary “drug holiday” from bisphosphonate therapy is recommended after about five years of oral use (or three years of intravenous use), since these agents have a long skeletal half-life; this strategy helps limit the risk of rare but serious complications such as atypical femoral fractures and osteonecrosis of the jaw [36]. To achieve the best results, it is important to plan Individualized treatment, educate the patient on the treatment plan, and monitor their progress [15]. Table 2 lists the types of drugs, their actions, common routes of administration, the conditions for which they are used for, and important safety and monitoring points. Each treatment includes references that support its use.

**Table 2:** Summary of pharmacological treatments for osteoporosis in elderly patients

Drug Class	Mechanism of Action	Administration	Indications	Important Considerations	References
Bisphosphonates	Inhibit osteoclast-mediated bone resorption by binding to hydroxyapatite in bone, reducing bone turnover, and increasing bone strength.	Oral (weekly/monthly), intravenous (yearly)	First-line therapy for postmenopausal osteoporosis and fracture prevention in elderly patients	Gastrointestinal irritation (oral forms), contraindicated in severe renal impairment, drug holidays recommended after long-term use to reduce rare adverse events such as atypical femoral fractures and osteonecrosis of the jaw	[16]
Alendronate, Risedronate, Zoledronic acid	Specific bisphosphonates are used with the above mechanism	Oral (weekly/monthly), intravenous (yearly)	Used in the treatment and prevention of osteoporosis and glucocorticoid-induced osteoporosis	Alendronate and risedronate orally; zoledronic acid intravenously; adherence and renal monitoring necessary	
Denosumab	Monoclonal antibody targeting RANKL, inhibiting osteoclast formation, function, and survival, thereby decreasing bone resorption	Subcutaneous injection every 6 months	Alternative for patients intolerant of bisphosphonates or with renal insufficiency; treatment of osteoporosis with high fracture risk	Risk of hypocalcemia, potential rebound bone loss upon discontinuation, requires calcium and vitamin D supplementation.	[23]
Anabolic Agents	Stimulate osteoblast activity, promoting new bone formation	Daily subcutaneous injection	Severe osteoporosis: patients who have failed or cannot tolerate antiresorptive therapies	Treatment duration is limited to 18–24 months due to the risk of osteosarcoma in animal studies, high cost, and the requirement for daily injections.	[12]
Teriparatide, Abaloparatide	Recombinant parathyroid hormone analogues that stimulate bone formation	As above	Approved for osteoporosis with high fracture risk, including glucocorticoid-induced osteoporosis	Close monitoring required; transition to antiresorptive therapy recommended after treatment cessation	
Selective Estrogen Receptor Modulators (SERMs)	Bind estrogen receptors, producing estrogen-like effects on bone while antagonizing estrogen effects in breast and uterine tissue.	Oral daily	Prevention and treatment of postmenopausal osteoporosis; reduction in vertebral fracture risk	Increased risk of venous thromboembolism, hot flashes, leg cramps; not effective for non-vertebral fractures	[28]
Raloxifene	A specific SERM used in postmenopausal osteoporosis	Oral daily	Approved for the prevention and treatment of osteoporosis in postmenopausal women	May reduce the risk of breast cancer; contraindicated in patients with a history of thromboembolism.	
Hormone Replacement Therapy (HRT)	Replacement of estrogen and progesterone to maintain bone density	Oral or transdermal	Selected postmenopausal women with osteoporosis and menopausal symptoms	Increased risk of cardiovascular events, breast cancer, and stroke limits long-term use; benefits must outweigh risks	[34]

Orthopedic implications of osteoporosis

Fractures in the hip, spine, and wrist, common with osteoporosis, greatly increase the risk of serious health problems and death among the elderly [3,37]. Orthopedic management of these fractures is challenging because poor-quality bones are more likely to have complications. Internal fixation or arthroplasty is normally used to treat hip fractures [31]. Weaker bones make it more difficult to gain stability with implants, resulting in screw loosening, implant failure, or poor fracture healing [36]. Orthopedic surgeons use specialized implants such as locking plates and intramedullary nails to help keep broken bones in place [38]. Proper preparation for bone health, such as correcting vitamin D deficiency or starting osteoporosis treatment, is very important for a successful surgery [39]. The most common type of osteoporosis is the vertebral fragility fracture. It can lead to pain, spinal deformity, and breathing problems [7]. Conservative care is often effective, but in severe cases, vertebroplasty or kyphoplasty may be needed to stabilize the fracture and reduce pain [29]. These procedures require careful planning as there are few implants available and there is a high risk for fracturing adjacent bones [38].

**Figure 2:**
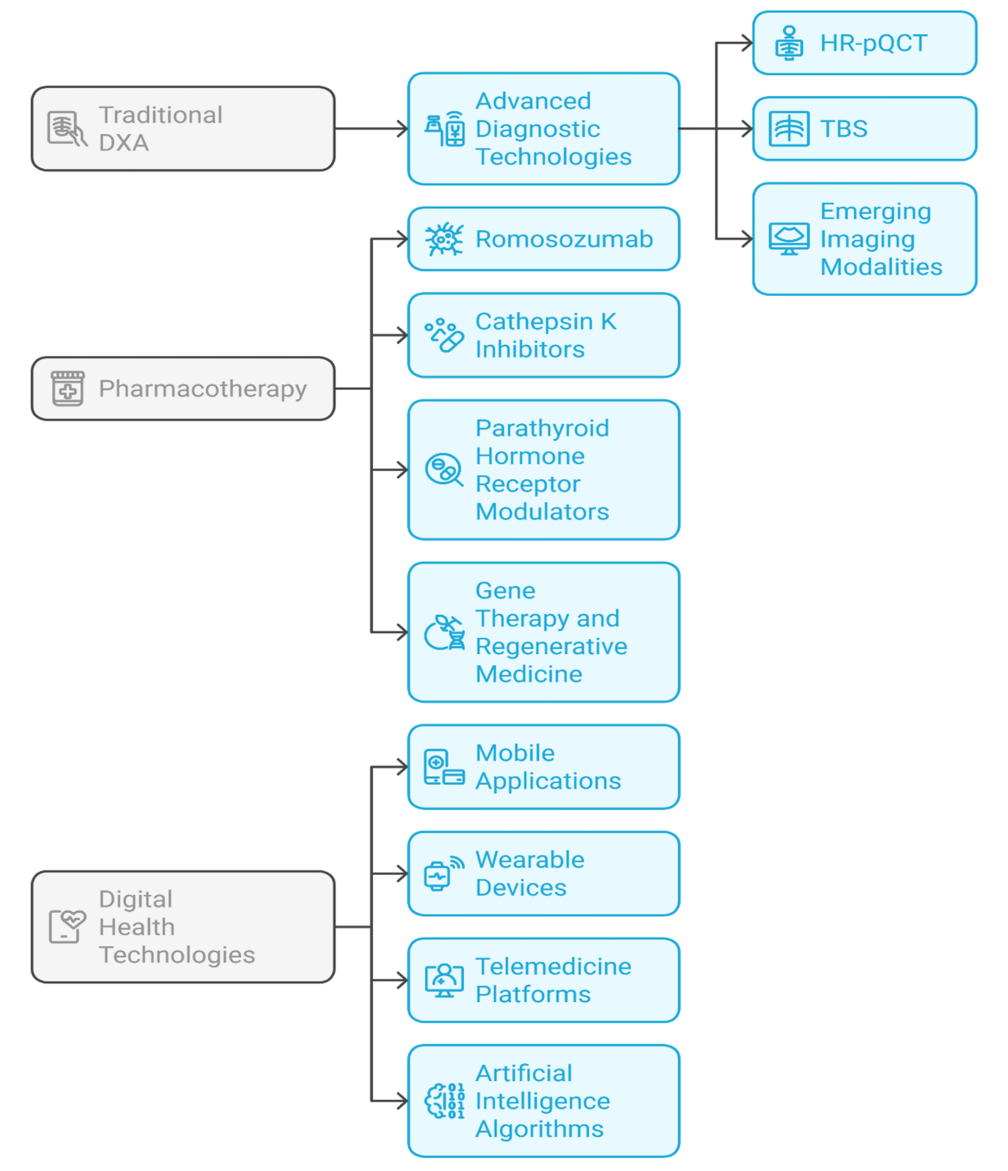
Framework of advancements in osteoporosis management Image credit: Loganathan T

Osteoporosis is often first noticed when a person has a distal radius fracture [21]. Casting or surgery are common treatments. Unfortunately, osteoporotic bone makes the treatment less predictable, and patients require longer rehabilitation [33]. Orthopedic surgeons are active in fracture prevention by working with other specialists in multidisciplinary fracture liaison services (FLS) [40]. The services help identify osteoporosis when a fragility fracture occurs, begin the right medical treatment, and organize rehabilitation, which helps prevent new fractures [37]. When feasible, early range of motion and weight bearing have been shown to improve recovery and minimize post operative complications [32]. Common osteoporotic fracture sites and possible treatments are shown in Figure 2, with emphasis on the body areas most likely to break and the surgeries used to treat them. Orthopedic care for osteoporosis is challenging and requires a combined approach of medical management, specialized surgery and integrated rehabilitation to improve patient outcomes [35].

Role of rehabilitation and physical therapy

Early rehabilitation within days to weeks of the fractures focuses assists in easing pain and early motion [41]. The main objectives of post-fracture rehabilitation are pain management, regain motion and strength, and prevent future fractures. Physical therapists help patients do exercises that increase their joint flexibility, strengthen their muscles, and improve their daily abilities [42]. This reduces risks from being immobile and enables patients to stay independent while doing tasks of daily living [43].

Moving your body through exercise can help build strong bones and prevent osteoporosis. Walking, climbing stairs, and resistance exercises should be done at least two or three times each week [15]. Tai chi, yoga, and certain balance exercises are important for preventing falls, improving balance, and reducing the risk of fractures [23]. A combination of physical therapy, nutrition advice, drug therapy, and mental support is essential for multidisciplinary care. Working together, orthopedic surgeons, physiatrists, dietitians, occupational therapists, and social workers help patients receive complete care. Patients need to learn how to move safely, change their environment, and avoid falls to reduce their risk of fractures [3,44].

**Figure 3:**
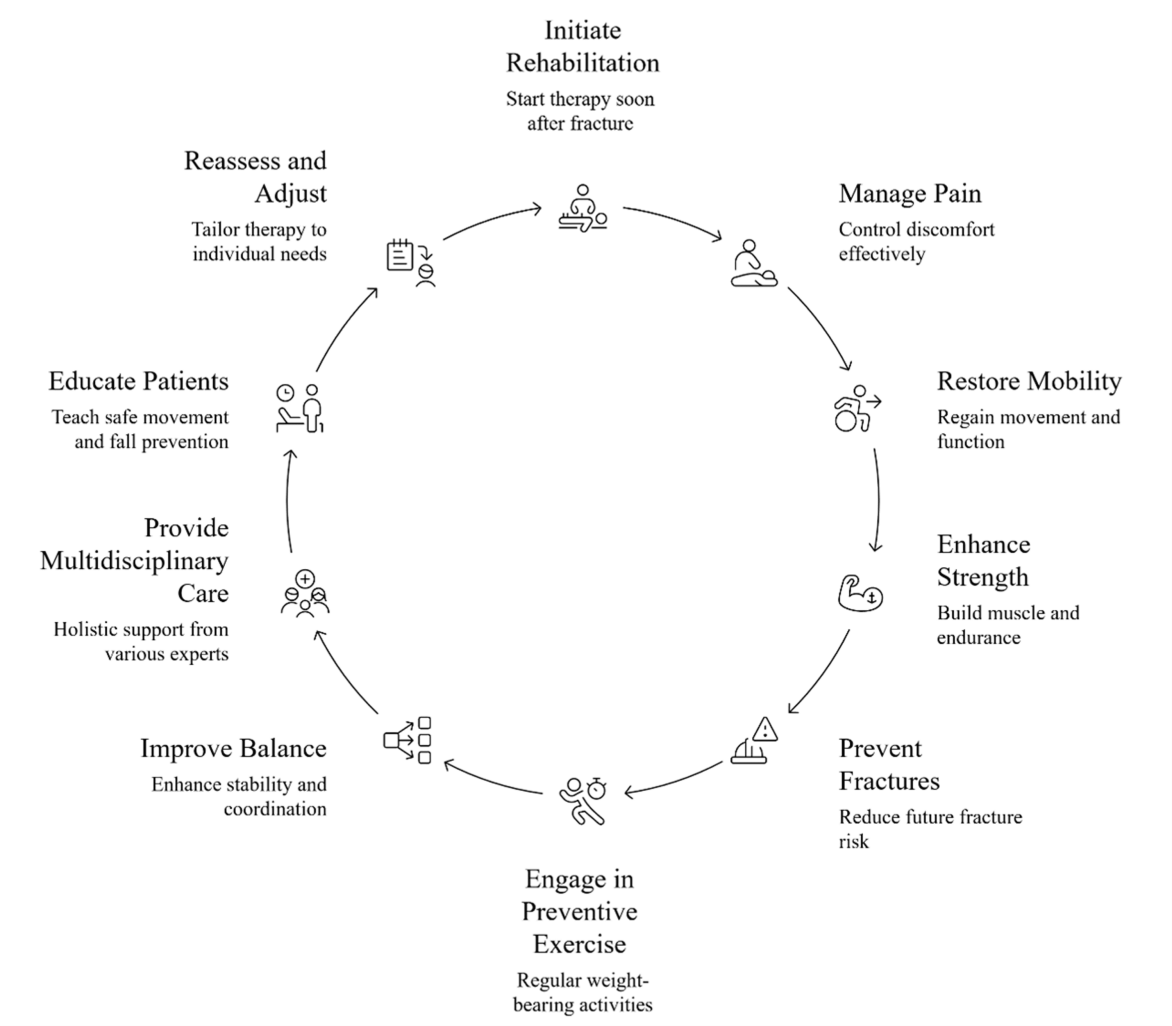
Cycle of osteoporosis rehabilitation and prevention Image credit: Loganathan T

When making a rehabilitation plan, so therapists make the plan-doctors make the referral to the therapist, doctors should take into account the patient’s baseline abilities, any other illnesses, and what the patient wants to achieve, and they should review and adjust the plan regularly [45]. Starting treatment early and continuing with rehabilitation has been proven to decrease hospital readmissions and improve how patients feel [22]. Rehabilitation and physical therapy are necessary for managing osteoporosis. Strong bones, better muscle function, and improved balance thanks to exercise help people recover from fractures and prevent further ones [14]. The circular model in Figure 3 outlines a continuous, patient-centered approach that begins with the early initiation of rehabilitation following a fracture.

Comorbidities and polypharmacy challenges

In elderly patients, osteoporosis often occurs in conjunction with conditions like diabetes, cardiovascular disease, and rheumatoid arthritis, these comorbidities influence bone health and fracture risk [7]. For example, people with type 2 diabetes often show preserved or even elevated bone mineral density (BMD), yet they experience higher fracture risk owing to compromised bone quality, including increased cortical porosity, accumulation of advanced glycation end products (AGEs), impaired bone microarchitecture, and this occurs independently of diabetic neuropathy or fall risk [21]. Also, cardiovascular diseases and osteoporosis have similar inflammatory patterns, and a few of their drugs may negatively affect bones or increase the risk of falling [33]. Because many patients now take several medications, polypharmacy can result in problems such as drug interactions and a lack of compliance with the treatment plan [25]. Some drugs, such as glucocorticoids and selective serotonin reuptake inhibitors, can lead to more bone loss, and a few may also make falls more likely [38]. To control osteoporosis in these circumstances, it is important to review all medications, remove any that may be harmful, and coordinate osteoporosis care with management of other chronic diseases [11]. The goal is to support healthy bones while reducing the risks caused by other diseases and their treatment [1]. Table 3 highlights the challenges in osteoporosis management associated with comorbidities and polypharmacy.

**Table 3:** Clinical challenges in osteoporosis management associated with comorbidities and polypharmacy

Challenge	Clinical Action	Goal	References
Multiple comorbidities	Screen for common diseases influencing bone health	Identify and address factors increasing fracture risk	[29]
Bone quality impairment in diabetes	Monitor beyond BMD with functional and fall risk assessments	Prevent fractures despite normal BMD	[21]
Medication-induced bone loss	Regular medication review and deprescribing, where possible	Reduce bone fragility and adverse drug effects	[15]
Polypharmacy-related fall risk	Minimize sedatives, optimize antihypertensives, and monitor hypoglycemic agents.	Lower fall and fracture incidence	[25]
Patient adherence difficulties	Educate patients and caregivers on medication importance and its side effects.	Improve long-term treatment adherence	[20]
Coordinated care needed	Multidisciplinary team approach including specialists, pharmacists, and therapists	Enhance comprehensive management and outcomes	[5]

Patient education and adherence to treatment

Teaching patients about osteoporosis is crucial, as the disease is often not noticed in elderly people until they break a bone [25]. If patients do not understand osteoporosis, they might not see the seriousness of the condition and may not follow their treatment plan properly [32]. These programs try to inform people about how osteoporosis can go unnoticed, the chances of fractures, and what can be done to manage the disease safely [29]. To ensure patients understand and engage with information, communication needs to fit their abilities, thinking skills, cultural backgrounds, and preferences [21]. Using a variety of educational aids, such as pictures, brochures, group sessions, and technology, helps patients understand their care and become involved in it [34]. The presence of family members and caregivers can help improve adherence and lifestyle changes, mainly in cognitively impaired people [23].

Adherence to osteoporosis medications is challenged by multiple factors. Gastrointestinal problems with bisphosphonates and the possibility of rare osteonecrosis of the jaw are common reasons why patients stop taking these drugs [22]. Patients find it difficult when they have to fast or stay upright after taking their medication [31]. The common issues of memory loss and thinking problems in the elderly make it harder for them to stick with their treatment plans when managing several medications. Cost and difficulty in accessing healthcare create obstacles for patient compliance [33]. Ways to help people take their medicines include giving them larger doses less frequently, using pill organizers or alarms, and having someone else help them take the drugs [30]. Following up with patients regularly helps doctors check the success of treatment, control side effects, and remind them to keep taking their medicine [24]. When both the clinician and patient discuss and consider the patient’s preferences, there is a greater chance the patient will accept and continue the treatment [27]. Patient support groups and community programs also give patients social motivation and useful advice, helping them keep taking their medicine [28]. Teaching patients about their condition and using specific interventions to help them stick to their treatment is important for better outcomes, fewer fractures, and a better quality of life in elderly people with osteoporosis [20]. Osteoporosis care works best when patients, caregivers, and healthcare professionals all work together [35].

Recent advances and innovations in osteoporosis management

The management of osteoporosis has improved a lot due to new technologies, different medications, and digital tools that help patients stick to their treatment [12,19]. DXA, which was previously the main way to measure bone mineral density, has been expanded by methods that look at bone quality and structure in greater detail [21]. HR-pQCT gives detailed information about bone structure in three dimensions, allowing for a better assessment of fracture risk than BMD alone, even if it is not widely available due to its cost and the skills needed to use it [14]. In addition, trabecular bone score (TBS) is an extra measure that, along with DXA, assesses the tiny structure of trabecular bone and can more accurately predict fracture risk without depending on BMD scores [28]. Magnetic resonance imaging (MRI) and quantitative ultrasound are being assessed as possible ways to assess bones without using radiation [26].

With the addition of agents such as romosozumab, which blocks sclerostin to increase bone formation and decrease bone loss, pharmacotherapy has advanced [17]. Studies have found that it improves bone density and reduces fracture risk. There are concerns about cardiovascular safety have affected how it is approved and used [20]. Cathepsin K inhibitors are another promising therapy as they stop osteoclasts from breaking down bone. New parathyroid hormone receptor modulators may help build bone with fewer side effects [22]. Gene therapy and regenerative medicine are promising ways to help bone regeneration but further studies are needed to establish their efficacy and safety [27].

Digital health technologies are creating new chances for patients to stay engaged and be monitored remotely [23]. Mobile apps allow patients to keep track of their drugs, meals, and exercise, which helps them follow healthy habits [15]. Using wearable devices, users and clinicians can get real-time alerts about fall risks related to gait and balance [11]. Because of telemedicine, people in underserved areas can now more easily see osteoporosis specialists. AI is being used to analyze medical images and health records to identify those at greatest risk and guide their treatments [29]. Even with all these improvements, problems related to data privacy, differences in digital skills, and unequal access to technology prevent many from using the internet [7]. These innovations allow for more precise treatment of osteoporosis, allowing each patient's treatment algorithm to meet their specific need [30].

Guidelines comparison and global perspectives

While there is international consensus to screen and treat osteoporosis, their implementation varies based on cultural and financial priorities [37,44]. Many guideline recommendations are based on the WHO’s FRAX tool, which combines clinical risk factors with or without BMD results [39]. NOF advises that postmenopausal women aged 65 and over, as well as men aged 70 and above, should be screened regularly, and treatment is suggested for those with prior fractures or a high FRAX score [41]. The IOF recommends using lifestyle changes, medications, and involving several healthcare professionals in caring for people with osteoporosis [35]. Even though there is agreement on basic ideas, the guidelines use different screening levels, age limits, and ways to rank risk factors [43]. For example, in some European countries, people with a high number of risk factors are screened earlier, as this is supported by the local rate of fractures and the healthcare services available [45]. In these areas, the main focus is on calcium and vitamin D supplements and preventing falls since access to tests and medications is limited [38]. They demonstrate that guidelines must be adjusted to meet the needs of each region.

Following osteoporosis guidelines is difficult in many parts of the world. Gaps between guidelines and actual practice occur because many healthcare providers and patients are not aware of the guidelines, the lack of DXA machines, the high cost of medication, and difficulties in adherence to treatment plans [46,47]. With the help of fracture liaison services, it is now easier to find and manage patients after a fracture, which makes it simpler to follow guidelines and organize joint care [36]. A summary of the main recommendations from WHO, NOF, and IOF points out differences in how they screen for osteoporosis, diagnose it, set treatment limits, and choose drugs for treatment [48]. This summary helps clinicians work with various guidelines and adapt their care to meet the needs of patients. Since the world’s population is aging, it is important to match these guidelines to the needs and medical resources of each community. Better education for both clinicians and patients, tackling major healthcare problems, and using new technology will play a key role in improving how osteoporosis is managed globally [49].

Limitations of the current evidence

At present, many barriers prevent the use of osteoporosis management in everyday clinical settings. Because study designs, the people involved, and the results measured can differ, it is difficult to combine findings and make them widely applicable. Many clinical trials do not include elderly patients with multiple health problems, so the results may not be useful for those who often have osteoporosis. The lack of long-term information about new therapies and advanced diagnostics means that many are reluctant to adopt them [9]. Although DXA is the most common way to measure bone mineral density, it does not show bone quality and important changes in bone structure, which are best seen with specialized imaging techniques that are not widely available in many clinics. Moreover, there are still problems with how well clinical trial results match what happens in real-world settings, which can influence patient outcomes.

Current guidelines for the treatment of osteoporosis have significant differences. This makes it difficult to decide on the best approach [50]. Many guidelines do not adequately address how to manage patients. The lack of clarity and consistency impedes implementation in poorer countries. Many protocols do not give enough attention to helping patients follow their treatment and understand it. Finally, it takes a long time to add new evidence and innovative approaches to guidelines, which may slow down the adoption of improvements in patient care.

Future recommendations

To solve the problems in treating osteoporosis, we need both research and new clinical approaches. Strategies are needed to rebalance treatment for osteoporosis in patients with chronic conditions. More studies are needed to evaluate technology and therapeutics used to treat osteoporosis in order to improve outcomes. Treatments should be centered on multidisciplinary teams to match each patient. Reliable algorithms for handling difficult cases and better fracture liaison services can lead to smoother care and more prevention. Treatment success continues to require patient education, help with adherence, and sharing decisions with patients [31]. Incorporating genetic, biochemical, and imaging data together makes personalized medicine a promising approach to tailoring treatments. This approach can also use predictive analytics and artificial intelligence to improve the accuracy of the risk of a fall and in selecting therapy. Partnerships among researchers, medical experts, and patients are necessary for creating treatments that address both the disease aspects and what matters to patients, making osteoporosis care better.

## Conclusions

Successful management of osteoporosis in the elderly requires a holistic approach that combines up-to-date evidence from various diagnostic techniques, pharmacotherapy, non-pharmacological interventions, and orthopedic management adapted to aging bone complexity. Guidelines adopted from bodies like the National Osteoporosis Foundation and the International Osteoporosis Foundation provide useful templates but often require tailoring for patients with complex comorbidities and polypharmacy. Early detection with the use of tools such as FRAX and supplementary measures such as trabecular bone score, accompanied by personalized treatment strategies, is key to enhancing fracture risk assessment and clinical outcomes. Orthopedic management, including fracture fixation and rehabilitation, continues to be pivotal in morbidity reduction and function preservation in this high-risk group.

In the future, the combination of new therapeutics like ramucirumab, imaging technologies, and digital health technologies, such as telemedicine and artificial intelligence-based risk prediction, is likely to increase the precision of osteoporosis care. It will be critical to have multidisciplinary coordination between rheumatology, endocrine, women’s health, ortho, and primary care with active patient participation and education to maximize adherence and long-term outcomes. Adopting these holistic, evidence-based approaches will be crucial in minimizing the global burden of osteoporotic fractures and enhancing the quality of life among older patients.
